# Reliability, interrelationships, and minimal detectable changes of strength and power metrics among well-trained rugby sevens players

**DOI:** 10.5114/biolsport.2024.133000

**Published:** 2024-02-08

**Authors:** Jad Adrian Washif, Kim Hébert-Losier, Nicholas Gill, Mazwan Zainuddin, Nur Sulastri Nasruddin, Ahmad Zawawi Zakaria, Christopher Martyn Beaven

**Affiliations:** 1 Sports Performance Division, Institut Sukan Negara Malaysia (National Sports Institute of Malaysia), Kuala Lumpur, Malaysia; 2 Division of Health, Engineering, Computing and Science, Te Huataki Waiora School of Health, University of Waikato, Tauranga, New Zealand; 3 All Blacks, New Zealand Rugby, New Zealand

**Keywords:** Explosive power, Monitoring, Rugby, Strength and power, Testing and measurement

## Abstract

Despite the importance of strength and power in rugby skills and match outcomes, there exists a noticeable gap in the measurement consistency and estimation of a true change of typical assessments designed to assess these qualities. To address this gap, we investigated the between-session reliability, interrelationships, and minimal detectable changes (MDC) of commonly used strength and power measures in team sports. Sixteen national-level rugby 7 s players were tested on two occasions, one week apart. Both the best and average (of 2–3 trials) peak force, peak power, height, distance, and/or strength indices during countermovement jump (CMJ), drop jump (DJ), isometric mid-thigh pull (IMTP), plyometric push-up (PPU), and standing long jump (SLJ) were obtained. Furthermore, one-repetition maximum (1RM) strength for bench press and back squat, reactive strength index, and dynamic strength index were also determined. Reliability was assessed using intraclass correlation coefficients (ICC) and coefficients of variation (CV), and used for MDC calculations, and interrelationships between variables were determined using correlation coefficients. Reliability was *excellent* for bench press, back squat, and SLJ (ICCs > 0.91); *high to excellent* for IMTP peak force, all CMJ, and DJ (except best DJ height and contact time), and PPU peak force parameters (ICCs > 0.78), with < 10% CVs (except PPU peak power). MDCs were generally smaller for average than best values. *Large to very* large relationships (r = 0.60 to 0.85) were observed between bench press, back squat, and IMTP with selected parameters of CMJ and PPU (p < 0.05), but not in DJ and SLJ. In conclusion, selected measures of strength and power displayed *high to excellent* reproducibility, with average values (rather than best) offering more stable assessments, and “smaller” MDCs. Based upon the relationships, it can be inferred that maximising strength would likely contribute to enhanced explosive performance.

## INTRODUCTION

The capacity to generate force (strength) is seen as a “driving vehicle” to maximising sports performance [1, 2] as sufficient strength levels enhance the ability to sprint, jump, throw, and engage in rugby-specific movements (e.g., tackles, rucks, and mauls). In support of this, significant correlations have been reported between lower body strength (as measured through squats) with tackling [3] and sprinting [4] abilities among rugby players. Notably, sprint speed itself has been linked to game-changing outcomes like line breaks, tackle breaks, and tries scored in elite-level rugby [5] and rugby sevens [6]. As these qualities directly contribute to the likelihood of winning rugby matches, assessing strength and power has become a standard part of players’ preparation. Indeed, these assessments facilitate the formulation of effective training regimens and allow for the monitoring of changes in an athlete’s physical condition. However, despite the critical role of strength and power in influencing rugby skills and match outcomes there remains a substantial gap in understanding how coaches and athletes can fully benefit from the typical assessments that focus on these attributes, especially for well-trained rugby players.

Currently, reliability and minimal detectable change (MDC) for common maximal strength assessments in rugby players, like bench press and back squat exercises, are scarce [7]. The MDC is defined as a valid change in score that is not due to chance. In sports, understanding MDC is crucial for accurately assessing the effectiveness of training programs, evaluating individual athlete progress, and distinguishing meaningful performance changes from random variability. In non-athletic populations, moderate and good reliability was reported for dynamic strength (e.g., squat), with Intraclass Correlation Coefficients (ICC) ranging between 0.64 and 0.99 (median ICC = 0.97), and Coefficients of Variations (CV) ranging between 0.5 and 12.1% (median CV = 4.2%) for bench press, back squat, and other strength exercises [7].

In contact sports like rugby, critical elements for successful performance include athletes’ stretch-shortening performance and musculotendinous unit capacity [8]. These are represented by tasks like plyometric push-up (for upper-body power), standing long jump (for horizontal power), drop jump (for reactive strength), and countermovement jump (CMJ, for vertical power) [9]. Development of these explosive qualities needs to consider maximal strength development [1, 10]. Maximal strength is defined as the highest load that can be lifted for one repetition (one-repetition maximum; 1RM). Even though it is practical, 1RM assessment may be difficult for new athletes owing to safety implications or when assessing a large group of athletes. In contrast, isometric strength assessment like the isometric mid-thigh pull (IMTP) appears more “realistic” for inexperienced athletes testing maximal strength, as it requires minimal technical advice [11]. Moreover, peak force during IMTP has been correlated with bench press and back squat exercises [12], and field-based tasks such as sprinting and change of direction [13]. When the IMTP is used in combination with the CMJ, it is possible to derive additional metrics, such as the dynamic strength index [14], which has been demonstrated to be a useful performance index in strength and conditioning training, research, and clinical settings [12, 14].

Even though essential, most of the previous studies assessing reliability and MDC have not considered well-trained athletes, like National-level rugby sevens players [14]. In addition, there are few studies that examine strength and power assessments (such as the IMTP, CMJ, and reactive strength index) by comparing the best to the average performance, particularly in well-trained cohorts [15]. Such information would ascertain whether the former or latter is more responsive or sensitive to changes in performance [15]. These limitations restrict team sport players and practitioners in the interpretation of whether or not the improvements from training are meaningful. Knowledge in the stability and precision of a measurement is fundamental to provide some level of confidence to practitioners (coaches, athletes), i.e., to effectively monitor training responses. For example, how much improvements in back squat is considered changes due to training effects? With this in mind, the present study aimed to investigate reliability, interrelationships, and MDCs for the typical strength and power assessments used in team and strength-power dependent sports in a National-level rugby sevens team. We hypothesised that maximum strength during dynamic and isometric tasks, as well as explosive power, and reactive strength, would exhibit high reliability and minimal MDCs, while the dynamic strength index would depend on the reliability of the CMJ and IMTP.

## MATERIALS AND METHODS

### Participants

Sixteen (n = 16) male national rugby sevens players (height 175 ± 5.2 cm; body mass 82.6 ± 7.6 kg, age 21.2 ± 2.1 y) were recruited. Most of the players (75%) were new to the National squad but had competed regularly at national-level competitions (> 5 years). Due to missing attendance, a smaller subset of participants (n = 11) completed the morning re-test session (all attended the afternoon session). Before agreeing to participate in the study by completing an informed consent form, each participant was given information about the risks and benefits of the study. This study was conducted in accordance with the declaration of Helsinki and approved by an Institutional Review Board.

### Study Design

**FIG. 1:**
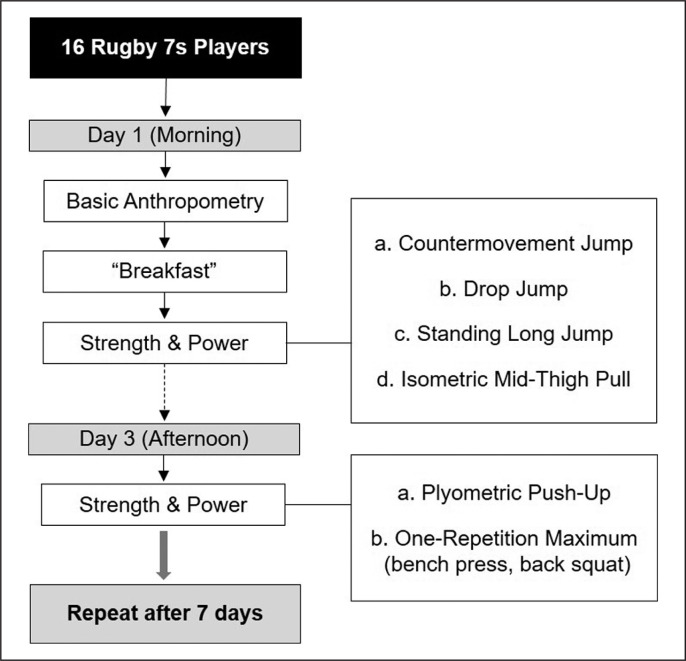
Schematic representation of data collection.

Test-retest reliability was conducted. Athletes were tested twice (two sessions each), a week apart. The second re-test session was conducted at the same time of day to reduce the impact of the time of day (Figure 1).

### Testing

The players had one full rest day prior to each testing session. Prior to testing, a standardised warm-up was conducted involving both general and specific warm-up [10]. The protocols used for the 1RM, IMTP, CMJ, drop jump, and plyometric push-up tests followed the recommendations and protocols from previous studies [16, 17, 18, 19, 20]. Except for the 1RM test (one attempt), the best and average performance of three attempts (two attempts for IMTP) were analysed for all assessments.

### Countermovement jump, drop jump, and standing long jump

The CMJ, drop jump, and standing long jump were used to assess leg power and reactive strength. Three trials were given for each test, with ~30–60 seconds rest interval between trials. When performing the CMJ, the players were instructed to stand up straight with their hands on the waist (akimbo). Upon “jump” instruction, they squatted down to knee angles of ~80–90°, jumped upward, and landed around the same standing points [20]. The players were asked to “jump as high as possible”. During the drop jump, the players were required to step-off (without sink down lower) a 40-cm box with their hands akimbo, land on both feet (forefeet area), and vigorously jump upwards [10]. The players were instructed to “minimise ground contact, and maximise jump height” while keeping upright body position (no tucking) during the jump, and landing with both feet on the same starting points [21].

Both CMJ and drop jump assessments were conducted using a force plate (400 series, Fitness Technology, Australia) sampling at 600 Hz, integrating the Ballistic Measurement System and software (BMS, Fitness Technology, Australia). The following variables were recorded (a) CMJ: concentric peak force, peak power, and jump height; (b) drop jump: contact time, jump height, and reactive strength index.

The reactive strength index was obtained during a drop jump using the ratio of jump height to ground contact time, (jump height / contact time). Flight time was utilised to calculate jump height, based on the equation of uniform acceleration, which stipulates that from the moment take-off was completed until subsequent ground contact upon landing, i.e., JH = 9.81 × (FT × FT)/8 [10, 20].

The standing long jump was conducted in a long jump pit. The standing long jump was initiated with both feet shoulder-width apart. The players were allowed to perform pre-stretch movement to a self-selected depth and swing their arms. A tape measure was used to record standing long jump distance (to the nearest 1 cm). The variable measures recorded were the absolute and relative (distance divided by stature) distance.

### Plyometric push-up

The players were instructed to begin the plyometric push-up at the top push-up position with their hands and feet on their respective force platforms, with a self-selected hand placement width. To perform a maximum plyometric push-up, they were then instructed to bend their elbows to a self-selected countermovement depth, and rapidly push upwards until their hands left the force platform [16]. To obtain vertical ground reaction force data for the plyometric pushup, each hand was placed on a force plate (device, *see* above), and each foot was placed on another force plate positioned at the same height. Three trials were performed, with ~30–60 seconds rest interval between trials. The variable measures recorded for the plyometric push-up included absolute and relative (divided by body mass) peak force and peak power.

### Maximum isometric strength

After the jump tests, the players performed a series of IMTPs, using a force plate (*see* above), with a custom-made rack designed to mount the bar at a specific height. For the warm-ups, the players performed IMTP once at ~50% effort, once at ~75% effort, and once at ~90% effort with 1 min rest interval. Subsequent to this, two IMTPs at maximum effort were performed, with a rest interval of 2–3 mins. During IMTP testing, the device was set to place the athlete in a typical power clean or pulling position that permits body position with the immovable bar around the mid-part of the thighs, while keeping the knee and hip angles at ~130–140° and ~145°, respectively [18, 19].

A 2D motion analysis software (Coach’s Eye, TechSmith Corp) was used to establish appropriate knee and hip angles (during the warm-up trials) to determine the height of the IMTP bar. The players were instructed to pull the bar “as quickly and explosively as possible” for 3–4 seconds with the “legs exerting maximally (fast and hard) against force plates,” after a countdown (“3, 2, 1, push”) was given. Two trials were recorded, with ~2–3 mins rest intervals. The resultant force-time data on a computer screen (connected to the force plate) was visually monitored to confirm appropriate actions (e.g., no countermovement), or otherwise, the trial was repeated. The variable measures recorded during the IMTP were absolute and relative peak force. Dynamic strength index was also calculated by dividing absolute CMJ peak force with the absolute IMTP peak force [17, 18].

### Maximum dynamic strength

Bench press and back squat exercises were used to conduct the 1RM strength test. For both exercises, the test was preceded by a warmup set of 10 repetitions using an unweighted barbell (20 kg). Subsequent to this, the players performed 10 repetitions, 5 repetitions, and 1 repetition (respectively) at 50%, 70%, and 90% of the expected 1RM. Next, 2 (minimum) to 5 (maximum) single attempts were made by raising (2–10 kg for bench press, and 5–20 kg for squat) or reducing (2–5 kg for bench press, and 5–10 kg for squat) the weight until the largest load that could be lifted correctly (for both exercises) was identified [10]. A rest period of 2–5 minutes was provided between each attempt. For back squat, an acceptable method consisted of lowering a weighted barbell to a depth that corresponded to knee angles of 80–85° as determined by a 2D motion analysis (Coach’s Eye, TechSmith Corp). Relative strength was also calculated for both exercises as 1 RM / body mass [10].

### Statistical Analyses

We calculated both relative and absolute reliability metrics. Relative reliability was assessed using ICC, which focus on the rank order or position of individuals within a group across repeated measures. Absolute reliability was evaluated using the Standard Error of Measurement (SEM), CV, and MDC, which measure the consistency of scores for the same individual across repeated tests, irrespective of their rank within the group. Relative reliability was rated as *poor, moderate, high*, and *excellent* based on ICC thresholds of < 0.50, 0.50, 0.75, and 0.90 [22]. For absolute reliability, CV values < 10% were considered to reflect acceptable absolute reliability. Absolute consistency was quantified using Bland–Altman statistics. The limits of agreement were calculated for both the lower (mean difference – (1.96 × SD)) and upper (mean difference + (1.96 × SD)) limit of agreement. MDC was calculated based on the ICC value, using an equation: MDC = 1.96 × SEMx√2. Relationships between explosive power and reactive strength variables with maximum dynamic strength, isometric strength, and dynamic strength index were determined using Pearson’s correlation coefficients. Both the best and average (of two or three trials) values were considered in the analyses. The Holm–Bonferroni corrections were conducted to control for the family-wise error of the statistical significance (p < 0.004). The following criteria were adopted to interpret the magnitude of the correlation: trivial (r < 0.1), small (0.1 ≤ r < 0.3), moderate (0.3 ≤ r < 0.5), large (0.5 ≤ r < 0.7), very large (0.7 ≤ r < 0.9), nearly perfect (0.9 ≤ r < 1), and perfect (r = 1) [23].

## RESULTS

All players completed all tests without incident, with data summarised in Table 1 and Table 2. Briefly: **(a)** *maximum strength. Excellent* reliability was found for 1RM bench press and back squat (ICCs: > 0.96, and CVs: ≤ 3.6%). *High* reliability was observed for best IMTP peak force and average IMTP peak force (ICCs: > 0.78, and CVs: ≤ 8.4%); **(b)** *vertical explosive and reactive strength. High* to *excellent* reliability was observed for all CMJ parameters (ICCs: 0.893–0.943 and CVs: 2.9–3.9%), as well as for drop jump parameters (ICCs: 0.812–0.942 and CVs: 3.6–7.3%); **(c)** *horizontal explosive strength. Excellent* reliability was observed for standing long jump parameters (ICCs: > 0.91, and CVs: ≤ 3.7%); **(d)** *upper-body power. Excellent* reliability was observed for plyometric push-up peak force parameters (ICCs: > 0.92, and CVs: ≤ 3.6%) but *poor* to *moderate* reliability was observed for plyometric push-up peak power parameters; **(e)** *dynamic strength index. Poor* to *moderate* reliability was observed for the dynamic strength index parameters.

**TABLE 1:** Performance scores, reliability measures, and minimal detectable changes for maximum dynamic and isometric strength, and dynamic strength index. Data are from male national rugby 7 s players.

	Mean, SD	ICC, ^D^_(95% CI)_	SEM _(95% CI)_	CV %, SD	SWC_0.5_	MDC %	MDC
***Dynamic Strength** (n = 16)*							
1RM bench press (kg)	92 ± 14	0.960^E^_(0.882, 0.986)_	1.0_(4.2, 0.2)_	3.2 ± 3.0	6.8	2.6	2.9
1RM back squat (kg)	136 ± 21	0.957_(0.881, 0.985)_	1.7_(5.9, −0.9)_	3.6 ± 2.6	10.3	3.5	4.8

***Isometric Mid-Thigh Pull** (n = 11)*							
Peak force, best (N)	2870 ± 336	0.809^H^_(−0.139, 0.964)_	56_(408, 190)_	7.4 ± 3.1	169	5.4	155
Peak force, average (N)	2842 ± 332	0.785^H^_(−0.038, 0.961)_	34_(405, 273)_	8.4 ± 1.6	166	3.3	94

***Dynamic Strength Index** (n = 11)*							
Peak force, best (au)	0.73 ± 0.05	0.398^P^_(0.369, 0.805)_	0.05_(0.10, 0.03)_	7.4 ± 4.6	0.02	19.2	0.14
Peak force, average (au)	0.71 ± 0.05	0.523^M^_(0.290, 0.863)_	0.04_(0.00, −0.14)_	6.5 ± 4.8	0.02	13.8	0.10

*Note:* SD, standard deviation; ICC, intraclass correlation coefficient; CI, confidence interval (upper, lower); SEM, standard error measurement; CV, Coefficient of Variation; SWC, Smallest Worthwhile Change; MDC, minimal detectable change; ^D^ descriptor; ^E^ Excellent; ^H^ High, ^M^ Moderate; ^P^ Poor; 1RM, one-repetition maximum.

The CVs were acceptable for all assessments (< 10%) except for peak power metrics during the plyometric push-up (~30%). Average values produced relatively lower CVs than best values (Tables 1 and 2). Based on MDC results, average values consistently produced smaller MDCs as compared to best values (Tables 1 and 2).

**TABLE 2:** Performance scores, reliability measures, and minimal detectable changes of explosive power and reactive strength variables. Data are from male national rugby 7 s players.

	Mean, SD	ICC, ^D^_(95% CI)_	SEM _(95% CI)_	CV %, SD	SWC_0.5_	MDC %	MDC
***Countermovement Jump** (n = 11)*							
Peak force, best (N)	2073 ± 205	0.928^E^_(0.747, 0.980)_	40_(92, −22)_	2.9 ± 2.5	102	5.3	111
Peak force, average (N)	2005 ± 169	0.893^H^_(0.559, 0.972)_	32_(125, −0.2)_	3.2 ± 2.3	85	4.4	89
Peak power, best (W)	4724 ± 529	0.930^E^_(0.295, 0.985)_	69_(319, 122)_	3.9 ± 1.8	264	4.0	191
Peak power, average (W)	4650 ± 532	0.933^E^_(0.450, 0.985)_	52 _(305, 101)_	3.9 ± 1.8	266	3.1	144
Jump height, best (cm)	44.5 ± 6.3	0.938^E^_(0.769, 0.983)_	1.0 _(1.9, −1.3)_	3.4 ± 4.0	3.1	5.0	2.2
Jump height, average (cm)	43.2 ± 5.9	0.943^E^_(0.979, 0.985)_	0.7 _(2.0, −0.7)_	3.7 ± 3.1	2.9	4.4	1.9

***Drop Jump** (n = 11)*							
Contact time, best (s)	0.22 ± 0.02	0.817^H^_(0.341, 0.950)_	0.010 _(0.2, 0.2)_	4.5 ± 3.4	0.01	9.6	0.021
Contact time, average (s)	0.20 ± 0.02	0.828^H^_(0.350, 0.954)_	0.010 _(0.2, 0.2)_	3.6 ± 3.0	0.01	7.7	0.016
Jump height, best (m)	0.28 ± 0.04	0.821^H^_(0.382, 0.951)_	0.01 _(0.01, −0.05)_	7.3 ± 4.8	0.02	14.5	0.04
Jump height, average (m)	0.27 ± 0.04	0.857^H^_(0.496, 0.961)_	0.01 _(0.01, −0.03)_	6.2 ± 4.3	0.02	11.5	0.03
RSI, best (au)	1.41 ± 0.24	0.901^E^_(0.600, 0.974)_	0.04 _(0.00, −0.16)_	6.8 ± 3.4	0.12	8.2	0.12
RSI, average (au)	1.31 ± 0.23	0.942^E^_(0.795, 0.984)_	0.03 _(0.01, −0.09)_	5.0 ± 3.2	0.11	5.4	0.07

***Plyometric Push-Up** (n = 13)*							
Peak force, best (N)	1260 ± 178	0.926^E^_(0.768, 0.977)_	26 _(82, −20)_	3.6 ± 3.6	54	5.7	72
Peak force, average (N)	1195 ± 173	0.961^E^_(0.873, 0.988)_	13 _(52, 1)_	3.0 ± 2.5	52	3.0	36
Peak power, best (W)	900 ± 329	0.074^P^_(−2.673, 0.732)_	610 _(1179, −1214)_	29.7 ± 38.6	164	188	1692
Peak power, average (W)	704 ± 276	0.541^M^_(−0.589, 0.862)_	258 _(453, −557)_	30.5 ± 34.4	138	101	714

***Standing Long Jump** (n = 13)*							
Distance, best (cm)	242 ± 16	0.919^E^_(0.738, 0.975)_	3.7 _(15.9, 1.5)_	3.8 ± 2.8	8.3	4.2	10.2
Distance, average (cm)	238 ± 17	0.941^E^_(0.790, 0.983)_	2.7_(11.7, 1.2)_	3.0 ± 2.6	8.5	3.1	7.4

*Note:* SD, standard deviation; ICC, intraclass correlation coefficient; CI, confidence interval (upper, lower); SEM, standard error measurement; CV, Coefficient of Variation; SWC, Smallest Worthwhile Change; MDC minimal detectable change; ^D^ descriptor; ^E^ Excellent; ^H^ High; ^M^ Moderate; ^P^ Poor; RSI, reactive strength index.

**FIG. 2:**
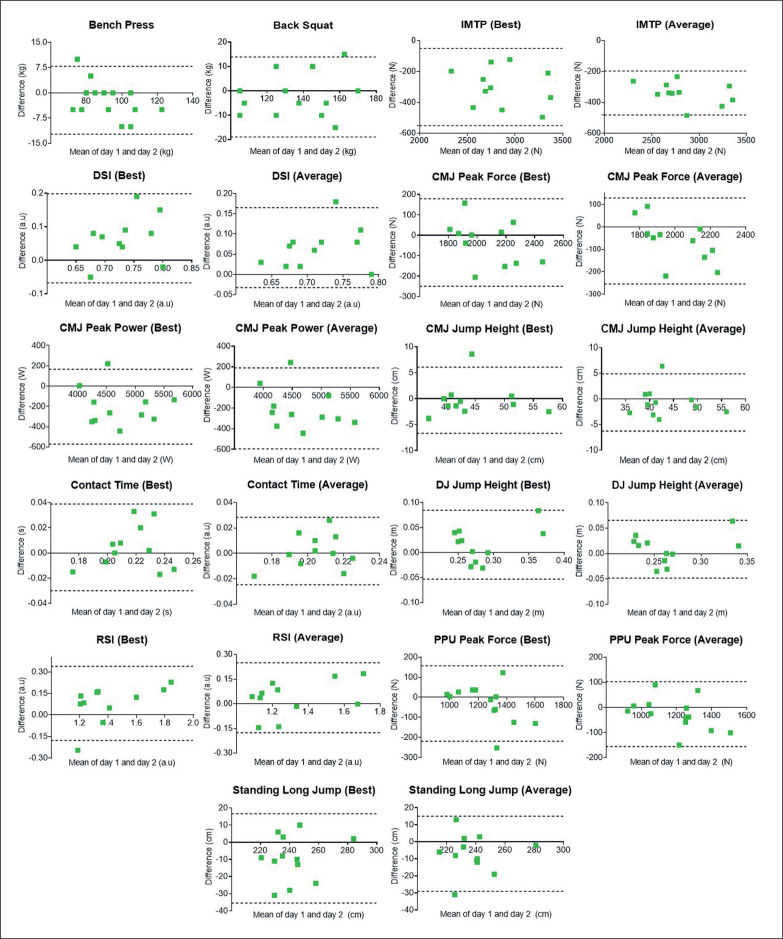
Bland-Altman limits of agreement (bias ± 95%) for strength and power assessments including best and average values. Note: IMTP, isometric mid-thigh pull; CMJ, countermovement jump; DSI, dynamic strength index; DJ, drop jump; RSI, reactive strength index; PPU, plyometric push-up.

Bland-Altman limits of agreement were employed to quantify the agreement between two paired variables, Day 1 and Day 2 assessments (Figure 2). Sub-figures show points (green squares) that are scattered and distributed (above and below zero), which suggests that there is no consistent bias or learning effect, except for the IMTP variables (Figure 2).

For the best value relationships, significant and *very large* relationships were detected between bench press (absolute or relative values) with CMJ peak force, and absolute and/or relative peak force during plyometric push up; as well as between IMTP with peak force during plyometric push-up. No significant relationships were observed between the maximum strength parameters with any parameters in drop jump and standing long jump. Additionally, significant and *very large* relationships were detected for bench press and back squat with IMTP peak force; and bench press with absolute and relative peak force in IMTP (Table 3).

**TABLE 3:** Relationships of best scores in maximum strength, with explosive power and reactive strength performance. Data are from male national rugby 7 s players (n = 14).

BEST	[1]	[2]	[3]	[4]	[5[	[6]	[7]
**[1]** 1RM bench press							
**[2]** Relative 1RM bench press	0.74**						
**[3]** 1RM back squat	0.78**	0.47					
**[4]** Relative 1RM Back Squat	0.38	0.60*	0.70**				
**[5]** IMTP peak force	0.78**	0.39	0.82**	0.35			
**[6]** IMTP relative peak force	0.71**	0.71**	0.63*	0.66*	0.69*		
**[7]** Dynamic strength index	-0.23	-0.09	-0.35	-0.21	-0.39	-0.27	

***CMJ***							
Peak force	0.72**	0.42	0.67*	0.29	0.82**	0.50	0.21
Relative peak force	0.28	0.38	0.30	0.33	0.38	0.46	0.59
Peak power	0.39	0.13	0.61*	0.39	0.63*	0.52	-0.23
Relative peak power	0.47	0.58	0.55	0.69*	0.49	0.62*	-0.01
Jump height	0.31	0.38	0.36	0.40	0.40	0.46	0.25

***Drop Jump***							
Contact time	0.04	0.33	-0.12	0.20	-0.47	-0.29	-0.10
Jump height	0.16	0.34	0.10	0.33	-0.11	0.14	0.48
Reactive strength index	0.02	0.07	0.02	0.10	0.05	0.24	0.43

***Plyometric Push-Up***							
Peak force	0.84**	0.79**	0.67*	0.36	0.73**	0.66*	-0.25
Relative peak force	0.47	0.81**	0.23	0.40	0.51	0.60	-0.35

***Standing Long Jump***							
Distance	0.08	-0.12	0.20	0.04	0.42	0.48	-0.13
Relative distance	0.14	-0.01	0.22	0.11	0.31	0.45	-0.06

* Significant at the 0.05 level (2-tailed);

** Significant at the 0.004 level (2-tailed). *Note:* 1-RM, one-repetition maximum; IMTP, isometric mid-thigh pull; CMJ, countermovement jump.

For the average value relationships, significant and *large* to *very large* relationships were observed for absolute and relative bench press with absolute peak force in plyometric push-up; and between absolute IMTP with CMJ peak force. No significant relationships were observed for the maximum strength parameters with any parameters in drop jump and standing long jump. Additionally, significant and *very large* relationships were observed for bench press with absolute back squat and IMTP peak force; between back squat and IMTP peak force (Table 4).

**TABLE 4:** Relationships of average scores in maximum strength, with explosive power and reactive strength performance. Data are from male national rugby 7 s players (n = 14).

AVERAGE	[1]	[2]	[3]	[4]	[5[	[6]	[7]
**[1]** 1RM bench press							
**[2]** Relative 1RM bench press	0.74**						
**[3]** 1RM back squat	0.78**	0.47					
**[4]** Relative 1RM Back Squat	0.38	0.60*	0.70**				
**[5]** IMTP peak force	0.74**	0.41	0.80**	0.33			
**[6]** IMTP relative peak force	0.45	0.52	0.49	0.62*	0.61*		
**[7]** Dynamic strength index	-0.30	-0.17	-0.37	-0.22	-0.48	-0.36	

***CMJ***							
Peak force	0.68*	0.36	0.67*	0.28	0.83**	0.52	0.09
Relative peak force	0.25	0.42	0.12	0.34	0.15	0.57	0.54
Peak power	0.39	0.13	0.62*	0.38	0.60*	0.38	-0.28
Relative peak power	0.42	0.51	0.49	0.61*	0.26	0.46	-0.05
Jump height	0.39	0.39	0.40	0.44	0.3	0.44	0.21

***Drop Jump***							
Contact time	0.13	0.37	0.00	0.27	-0.41	-0.29	-0.02
Jump height	0.16	0.33	0.07	0.28	-0.12	0.12	0.53
Reactive strength index	0.18	0.40	0.09	0.22	0.03	0.20	0.32

***Plyometric Push-Up***							
Peak force	0.85**	0.71**	0.60*	0.32	0.70**	0.50	-0.23
Relative peak force	0.19	0.66*	-0.11	0.30	-0.31	0.32	0.30

***Standing Long Jump***							
Distance	0.01	-0.15	0.13	-0.01	0.32	0.39	-0.24
Relative distance	0.09	-0.03	0.17	0.09	0.20	0.34	-0.14

* Significant at the 0.05 level (2-tailed);

** Significant at the 0.004 level (2-tailed). *Note:* 1RM, one-repetition maximum; IMTP, isometric mid-thigh pull; CMJ, countermovement jump.

## DISCUSSION

The findings of the current study indicate (i) *excellent* reliability for maximum strength in bench press, back squat, and standing long jump, all CMJ parameters (except average peak force, *high*), drop jump RSI, and peak forces during plyometric push-up; (ii) *high* reliability for peak force parameters during IMTP, and drop jump parameters (contact time and jump height); (iii) high levels of reliability can be achieved with one familiarisation session performed just prior to the actual assessment without inducing a systematic learning effect (except for IMTP) for these strength and power tests; (iv) acceptable levels of absolute reliability for all tests (CV < 10% except peak power during plyometric push-up); (v) average, rather than best values, optimised the stability of measurement, and provided a relatively smaller MDC; and (vi) significant and *large* to *very large* relationships between bench press and peak force during plyometric push-up and CMJ, but not in any parameters of drop jump and standing long jumps, for both best and average values. These findings offer practitioners with useful benchmarks to estimate whether the changes in performance (or progression) for strength and power variables can be interpreted as meaningful.

Dynamic and isometric strength are usually assessed in strength and conditioning setting. Between these two types of maximum strength tests, we found *excellent* reliability for the dynamic strength (bench press and back squat), whereas *high* reliability for the best and average peak force of IMTP. These findings might be attributed to the players’ history of training with bench press and back squat exercises, as well as their exposure to actual testing protocols during earlier testing sessions. Grgic et al. [7] reviewed 32 studies investigating the reliability of one-repetition maximum, and found generally *excellent* reliability of dynamic strength (various exercises) from a range of populations, but only two included team-sport athletes and these were either adolescents aged ~16 [24] or defined as inexperienced [19]. More recently, Grgic et al. [25] reported that IMTP maximum strength assessment has *good to excellent* test-retest reliability based on a review of the literature [25]. Like-wise, Aben et al. [26] reported acceptable between-day reliability for the IMTP peak force among professional male rugby players (n = 10). Based on the findings of the current study, it is possible to infer that dynamic and isometric strength may be used as reliable evaluations of maximal strength among athletes, albeit more familiarisation with IMTP is required if this exercise is not already part of athletes’ training.

The current study also found *excellent* reliability of various measures of explosive power and reactive strength. CMJ and drop jump exercises are usually thought to represent slow (i.e., CMJ) and fast (i.e., drop jump) stretch-shortening cycle, which are decisive in highspeed, short-event performance such as sprinting [10, 27]. Supporting the current study’s findings, earlier research has reported *excellent* reliability in CMJ variables (without specifying either best or average scores) among professional male rugby players [26]. Comparable to the current study [9], the authors found an acceptable level of reliability of the reactive strength index (ICC of 0.93, CV of 8.5%) among elite junior rugby players. CMJ height may lack sensitivity to detect neuromuscular changes in the context of Australian Rules football match [28], as compared to peak force and peak power [28]. As force plate CMJ analysis offers a greater range of kinetic and kinematic outputs including peak force, peak power, and flight time [10, 13], we assessed these parameters and found that specific metrics in CMJ (e.g., peak power and jump height) and drop jump (e.g., reactive strength index) were highly repeatable. Further, it is worth noting that the dynamic strength index showed relatively poor reliability among all the assessed variables in the current study. Dynamic strength index variables of IMTP and CMJ are assessed independently, and thus, subject to errors from different test occasions. Comfort et al. [14] stated that the dynamic strength index as derived from CMJ rather than squat jump is a more stable method of assessing the dynamic strength index. However, we observed that the dynamic strength index also depends on IMTP’s reliability measures (Table 1, Figure 2). Additionally, the explosive measure of upper body (plyometric push-up) revealed similar (reliable) findings for the peak force variables, but not peak power. Taken together, CMJ and drop jump metrics have been widely used in athletes’ monitoring and research, and these variables appear reliable. Nonetheless, the precision of the dynamic strength index can be limited as it relies on the collection of two reliable measurements for the data to be useful.

Practitioners should be aware that some assessments may be more sensitive when data are averaged rather than relying on best scores. Notably, we found that the reliability level was higher for average than best values, e.g., average reactive strength index (*excellent* vs *high*) and average IMTP peak force (*high* vs *moderate*). Roe et al. [15] suggest that CMJ mean power (taking maximal score from 2 or 3 attempts), peak force, or mean force and plyometric-push-up mean force (from 2 or 3 attempts) produced acceptable reliability (CV < 5%) and good sensitivity (CV < smallest worthwhile change) [15]. A lower CV for a test implies less random noise, and therefore a greater ability or likelihood of detecting a real change in performance [29]. Furthermore, an MDC greater than the CV of a test implies higher measurement error, which might limit the usefulness of specific tests [29]. Importantly, average results yielded relatively smaller MDCs compared to the best results. Comfort and McMahon [19] reported MDCs of approximately 5–6% in the back squat and power clean in inexperienced athletes, which is slightly larger than the current study (~3–5%). Thus, MDC calculation (best or average results) can help athletes and coaches set goals more appropriately. Employing MDC is necessary for athletes because any changes in strength and power measurements exceeding the MDC value can be deemed as real changes due to training rather than changes resulting from a random variation or errors in measurement.

High levels of reliability could be achieved in several strength and power tests with familiarisation sessions performed just prior to the actual assessment without inducing a systematic learning effect (confirmed by Bland-Altman plots). In this study, most players (> 75%) were new recruits in the national squad; and started to get exposure to typical tests in team sports. Importantly, the same test exercises (e.g., bench press, back squat, vertical jump) here were done regularly by the players during training, except for the IMTP and drop jump. The variation between trials may be considered to be intrinsic variation derived from independent sources of mistakes or random errors. Poor reliability might lead to different scores across two test administrations, and subsequent misinterpretation of change scores. Some exercises (e.g., vertical jump) can be performed without the need for familiarisation trials [30]. Given the challenge to conduct specific familiarisation sessions among elite athletes (usually, done only before the actual assessments as in the current study), high levels of reliability could be achieved in several strength and power tests, as noted. These findings agree with prior literature [14, 31]. However, additional familiarisation may be required for IMTP to improve the test-retest reliability.

The relationships between bench press with peak force during plyometric push-up and CMJ were *very large*. Training-induced increases in maximal strength can improve force generation and power production [2], with a transfer to greater athletic performance [2]. High levels of strength are often correlated with explosive performance, which may be linked to enhanced nervous system capacities e.g., rate of motor unit activation [1]. In the current study, *large* to *very large* relationships were found for dynamic and isometric strength with CMJ peak force, but not CMJ height. In accordance with Thomas et al. [32] who investigated team-sport athletes, absolute IMTP peak force does not necessarily correlate with CMJ height, but it does appear to correlate with absolute CMJ peak force and peak power. In the current study, this holds true especially for the best values observed. One possible reason for this observation is related to the overestimation of true flight time when using flight time to estimate jump height, as the jumps are not always performed uniformly with a consistent body position [20]. In contrast, McGuigan et al. [12] reported significant relationships between vertical jump height with bench press, back squat, and IMTP among recreational-level individuals, which indicates a discrepancy in findings due to different population, e.g., spread in dataset values and standard deviation [27]. There appears to be some unexplained variance between the maximum strength in back squat, IMTP, and bench press with drop jump, as well as standing long jump among this cohort. Plausibly, the aforementioned maximum strength exercises do not influence actions requiring more lower-limb muscles and joints (triceps surae and ankles) as stressed during drop jump, as well as force production in a horizontal manner (i.e., standing long jump). Mean-while, biomechanical similarities (joint actions) in the upper body between the plyometric push-up and bench press, appear to be supported by the significant and *very large* correlation between both exercises. This observation may also be related to the similarity of muscle contractions [16]. These relationships between maximum strength and explosive tasks imply that, a concurrent strength and power training program, may be appropriate for improving performance in explosive exercises.

This study is not without limitations. It is possible that more specific familiarisation sessions would have altered the observed outcomes, (e.g., smaller MDCs). However, the current study represents the situation encountered by any teams performing testing and measurement at the beginning of their training preparation and can be considered to be ecologically valid. Secondly, the sample size can be considered *small-modest* and was affected by the missing attendance of players during the re-test sessions, which might have influenced how the conclusion was drawn. In particular, a proportional bias and non-uniform scatter may exist in several sub-plots (Figure 2), issues likely amplified by our small sample size. Additionally, we acknowledge that small sample size can adversely affect the SEM, thereby directly impacting MDC values. Moreover, the effects of timing of different variables must be noted; power variables assessed in the morning and dynamic strength variables measured in the afternoon (although being maintained during the retest), which could introduce “time of day” effects that may influence the outcomes. The results must also be considered in the context of limitations associated with correlations, which do not demonstrate causality and effect. Future research employing larger sample sizes are needed to replicate and validate our findings, and investigate covariance or the impact of performance changes (same players) over time (during early and late preparation, and competition periods) on test-retest reliability and MDC values.

## CONCLUSIONS

In conclusion, a few commonly used tests in team sports, including bench press, back squat, standing long jump, and specific metrics (e.g., force, power) during IMTP, CMJs, drop jumps, and plyometric push-ups, showed *excellent* reproducibility, while exhibiting low variability (< 10% coefficient of variation). Furthermore, we found that dynamic strength index, and power metrics during plyometric push-ups presented *low* reproducibility. In evaluating the impacts of trials, we observed that the average score values, as opposed to the best score values, appeared to optimise measurement stability and produced comparatively lower MDCs. Furthermore, maximum strength was strongly associated with explosive performance, which suggests that muscular strength would markedly contribute to enhanced force and power output during explosive lower- (e.g., CMJ) and upper- (plyometric push-up) body.
